# Cardiovascular event risk estimated after coronary revascularization and optimal medical therapy: J-ACCESS4 prognostic study

**DOI:** 10.1007/s12149-020-01558-4

**Published:** 2021-01-03

**Authors:** Tomohiko Sakatani, Kenichi Nakajima, Hiroshi Fujita, Tsunehiko Nishimura

**Affiliations:** 1 Department of Cardiology, Japanese Red Cross Kyoto Daini Hospital, 355-5 Haruobi-Cho, Kamigyo-Ku, Kyoto, 602-8026 Japan; 2 Department of Functional Imaging and Artificial Intelligence, Kanazawa University, Kanazawa, Japan; 3 Graduate School of Medical Science, Kyoto Prefectural University of Medicine, 465 Kajiicho, Kawara-Machi Hirokoji, Kamigyo-Ku, Kyoto, 602-8566 Japan

**Keywords:** Cardiac ischemia, Single-photon emission computed tomography, ^99m^T_c_-tetrofosmin, Multicenter study

## Abstract

**Background:**

An assessment of cardiac events and survival using quantitative gated myocardial single-photon emission computed tomography (SPECT) (J-ACCESS) associated several risk factors with cardiac events in Japan. The clinical course after revascularization and/or optimal medical therapy (OMT) was followed in patients with coronary artery disease (CAD) at moderate-to-high risk estimated by software incorporating the J-ACCESS risk model. The present study aimed to determine the relevance of changes in estimated risk to outcomes of these therapies.

**Methods:**

This study included 494 patients with possible or definite CAD who underwent initial pharmacological stress ^99m^Tc-tetrofosmin myocardial perfusion SPECT (MPS) before and eight months after therapy. Major cardiac event risk during 3 years of follow-up was calculated using an equation based on that in the J-ACCESS study. Patients with ≥ 10% cardiac event risk estimated at the first MPS (*n* = 31) were analyzed and followed up for at least 1 year.

**Results:**

Estimated risk was reduced by ≥ 5% in 14 patients (45%) after therapy. During a follow-up period of 22.1 ± 6.7 months, one patient without such reduction had a major cardiac event. Mean %summed stress scores significantly decreased from baseline to follow-up in patients with and without risk reduction. Left ventricular ejection fraction (LVEF [%]) at rest was significantly increased at the second, compared with the first MPS between patients with, than without risk reduction (57 ± 17 vs. 45 ± 16%, *p* = 0.001 and 50 ± 11 vs. 49 ± 9%, *p* = 0.953, respectively).

**Conclusions:**

A reduction in cardiac ischemia and an increase in LVEF by revascularization and/or OMT were necessary to avoid cardiac events among patients with moderate-to-high estimated risk, and changes in event risk were quantifiable.

## Introduction

For several decades, percutaneous coronary intervention (PCI) has been the main therapeutic method for treating patients with coronary artery disease (CAD) associated with myocardial ischemia. Although PCI reduces the incidence of death in patients with acute or unstable coronary syndromes [1–3], the benefit of PCI to long-term prognoses compared with optimal medical therapy (OMT) has not been assessed in patients with stable CAD [4]. On the other hand, a higher ischemic burden contributes to cardiac death, myocardial infarction, and coronary revascularization due to worsening angina symptoms. A nuclear substudy of the COURAGE trial of 314 patients who underwent myocardial perfusion single photon emission-computed tomography (SPECT) before and after PCI found significant ischemic reductions in patients treated with OMT in addition to PCI [5]. The clinical course was favorable in patients who achieved at least a 5% reduction in ischemia after PCI [6, 7]. Other studies also indicated that a moderate-to-severe ischemic burden was worth treating by PCI, which could sufficiently reduce ischemic burden [8–10].

Myocardial perfusion SPECT (MPS) is now an established tool with which to diagnose CAD. The prognostic value of SPECT findings, whether normal or abnormal, has been confirmed in a large-scale multi-center observational study in Japan (Japanese Assessment of Cardiac Events and Survival Study by Quantitative-gated SPECT [J-ACCESS] [11]. Multivariate analysis in that study selected age, larger perfusion defects, reduced ejection fraction, larger ventricular volume, and diabetes mellitus as independent predictors of adverse cardiac events. Three-year cardiac event risk can be estimated based on the J-ACCESS risk model, which has been incorporated into Heart Risk View (HRV) software. This software calculates the probability (%) of major cardiac events (cardiac death, nonfatal myocardial infarction, and severe heart failure requiring hospitalization) arising during 3 years of follow-up [12–15]. The J-ACCESS4 study then clarified the prognostic impact of reducing myocardial ischemia among Japanese patients with stable CAD [6, 16]. That multicenter, prospective cohort study using electrocardiography (ECG)-gated MPS provided data about associations between ischemic reduction and prognosis in patients with stable CAD, but how to use the HRV software to estimate cardiac event risk was not clarified in the clinical setting. Thus, the present substudy of J-ACCESS4 assessed the clinical application of HRV by analyzing data from patients with moderate-to-high risk estimated by HRV and followed their clinical course after PCI and/or OMT. We postulated that HRV would predict cardiac events more accurately than MPS alone and generate more useful information about therapeutic effects.

## Methods

This study is a subanalysis of J-ACCESS4, the methods of which are described in detail elsewhere [6, 16].

### Study population

The study population of the J-ACCESS4 study comprised 494 patients registered at 59 institutions. The inclusion criteria comprised: age ≥ 20 years, scheduled to undergo stress-rest ECG-gated MPS due to possible or definite CAD, at least one cardiac symptom, and ECG changes indicative of CAD. The patients were treated by coronary revascularization and/or medications within 2 months after MPS and were assessed by a second stress/rest MPS 4–10 months later. Whether the treatment for each patient would be revascularization or medical therapy was judged by their attending physicians. Exclusion criteria comprised acute coronary syndrome within 3 months, prior diagnosis of myocardial infarction, hemodialysis, and severe congestive heart failure (New York Heart Association class III or higher). The presence or absence of cardiac events was followed in all patients for at least 1 year after the second MPS. All patients provided written informed consent to participate in all protocols associated with the study before enrolment. All procedures in the present study involving human participants complied with the ethical standards of each institutional research committee and with the principles of the Declaration of Helsinki (2013 amendment).

### Myocardial perfusion SPECT study

Stress-rest ^99m^Tc-tetrofosmin SPECT imaging proceeded at each hospital using a standard protocol [6, 16], and pharmacological stress was applied using adenosine. The SPECT acquisition protocols were not precisely regulated and depended on each institution. The patients were assessed using both stress and rest ECG-gated images. Gated SPECT findings were quantified using QGS software (Cedars Sinai Medical Center, Los Angeles, CA, USA) at all institutions. Imaging and image processing procedures were separately confirmed.

### Quantitative data analysis of perfusion SPECT

Short, vertical, and horizontal long-axis SPECT images were generated using a standard processing protocol that was verified by the J-ACCESS study [6, 11, 16]. All reconstructed short-axis data created at participating hospitals were sent to the J-ACCESS office (Osaka, Japan) in Digital Imaging and Communication in Medicine (DICOM) format. An image interpretation committee digitally evaluated submitted MPS images in a blinded manner [6, 11]. The SPECT images were divided into 17 segments, visually scored using a 5-point scale from normal (0) to defective (4), then further analyzed. The summed stress and rest scores (SSS and SRS) were calculated by adding the scores of these segments of the left ventricle. The summed differential score (SDS) was calculated as summed stress (SSS) minus summed rest (SRS) scores converted to ratios (%) of the total myocardium (%Myo stress, %Myo rest, and %Myo ischemia, respectively). The %myocardium was calculated as summed scores divided by a maximum score of 68, and a decrease in ischemia was calculated as the difference in %Myo ischemia between before and after treatment. Left ventricular ejection fraction (LVEF), end-diastolic (EDV) and end-systolic (ESV) volumes were analyzed using quantitative gated SPECT (QGS) software.

### Heart risk view

Heart Risk View software was created during 2007 to clinically evaluate risk of cardiac events. Several risk factors that J-ACCESS associated with cardiac events [11] were included in risk estimation. The Heart Risk View-S software (HRV-S) calculated the probability of major cardiac events, including cardiac death, non-fatal myocardial infarction, and hospitalization due to severe heart failure, occurring over a period of 3 years, using the following equation based on a multi-variable logistic model [12–15]:

$$ \begin{aligned} {\text{Three - year cardiac event risk}} \, \left( \% \right) & = {1}/\left( {{1} + {\text{Exp}}\left[ { - \left( {{-}{4}.{8125} + 0.{8858} \times {\text{diabetes}}\left( {0{\text{ or 1}}} \right) + 0.0{558} \times {\text{age}} \, \left( {{\text{years}}} \right) + 0.{1941}} \right.} \right.} \right. \\ & \left. {\left. { \times {\text{SSS category}}\left( {0,{1},{2},{\text{or 3}}} \right){-}0.0{475} \times {\text{LVEF}} \, \left( \% \right)} \right]} \right) \times 100. \\ \end{aligned} $$

This equation is incorporated into the myocardial SPECT image analysis tool of HRV-S (Nihon Medi-Physics. Co. Ltd., Tokyo, Japan).

### Patient follow-up

The primary endpoints were cardiac death, sudden death of unknown causes, non-fatal myocardial infarction, and hospitalization due to worsening heart failure. Only the first event was counted, even when several cardiac events occurred during follow-up.

### Statistical analysis

Variables are expressed as means ± standard deviation. Normally distributed parameters were compared using *t* tests, and non-normally distributed data were analyzed using Mann–Whitney *U* tests. Normality was determined using Shapiro–Wilk tests and categorical data were compared using Fisher exact tests. Linear regression lines were calculated between two variables using the least squares method. Values with *p* < 0.05 were considered statistically significant.

## Results

### Clinical characteristics of the patients

Among 494 patients, 31 with ≥ 10% probability of cardiac events at the first MPS were defined as being a moderate-to-high risk, and they were analyzed in a follow-up study. Table 1 shows the clinical characteristics of the patients (mean age, was 75.9 ± 8.3 years); 87%, 90%, and 81% of them had a history of hypertension, diabetes, and dyslipidemia, respectively. The %Myo stress and %Myo ischemia values at the first MPS assessment were 14.1 ± 9.2% and 5.6 ± 6.6%, and were categorized as moderate risk [11], and 68% of the patients were treated with statins for dyslipidemia.

**Table 1:** Characteristics of study population

Age, years	75.9 ± 8.3
Male gender, *n* (%)	25 (81%)
Cardiac risk factors, *n* (%)	
Hypertension	27 (87%)
Diabetes	28 (90%)
Dyslipidemia	25 (91%)
Peripheral arterial disease	2 (6%)
Current smoker	6 (19%)
Family history (cardiac diseases)	1 (3%)
Blood examination	
CRP, mg/dL	0.3 ± 0.5
HbA1c, %	6.8 ± 1.0
Cr, mg/dL	1.0 ± 0.3
eGFR, mL/min/1.73 m^2^	59.1 ± 21.2
LDL cholesterol, mg/dL	96.3 ± 21.8
HDL cholesterol, mg/dL	47.2 ± 14.7
TG, mg/dL	115.7 ± 53.7
Medications, *n* (%)	
ACE inhibitors	7 (23%)
ARB	11 (35%)
CCB	12 (39%)
Beta blocker	10 (32%)
Nitrate	11 (35%)
Aspirin	21 (68%)
Clopidogrel	11 (35%)
Statin	21 (68%)
Insulin use	6 (19%)
1st SPECT	
%Myo stress	14.1 ± 9.2
> 5%, *n* (%)	25 (81%)
> 10%, *n* (%)	21 (68%)
%Myo rest	8.4 ± 7.9
> 5%, *n* (%)	19 (61%)
> 10%, *n* (%)	12 (39%)
%Myo ischemia	5.6 ± 6.6
> 5%, *n* (%)	14 (45%)
> 10%, *n* (%)	10 (32%)
LVEF, % (rest)	47.2 ± 12.4
EDV, mL (rest)	117.0 ± 48.3
ESV, mL (rest)	66.5 ± 44.3
LVEF, % (stress)	44.6 ± 11.6
EDV, mL (stress)	118.5 ± 43.1
ESV, mL (stress)	69.5 ± 39.7
2nd SPECT	
%Myo stress	7.5 ± 7.8
> 5%, *n* (%)	15 (48%)
> 10%, *n* (%)	12 (39%)
%Myo rest	6.0 ± 6.6
> 5%, *n* (%)	13 (42%)
> 10%, *n* (%)	8 (26%)
%Myo ischemia	1.5 ± 3.6
> 5%, *n* (%)	4 (13%)
> 10%, *n* (%)	1 (3%)
LVEF, % (rest)	53.0 ± 14.3
EDV, mL (rest)	105.9 ± 41.7
ESV, mL (rest)	54.3 ± 35.6
LVEF, % (stress)	48.6 ± 13.5
EDV, mL (stress)	110.1 ± 41.0
ESV, mL (stress)	60.4 ± 35.9
Risk estimated by HRV (1st SPECT), %	17.2 ± 7.8
Risk estimated by HRV (2nd SPECT), %	8.3 ± 2.4
Change in risk estimated by HRV, %	4.5 ± 5.3
Cardiac event, *n* (%)	1 (3%)
Revascularization, *n* (%)	22 (71%)

*%Myo ischemia* ratio of ischemic myocardium (from summed difference score), *%Myo stress* ratio of hypoperfused myocardium under stress, *%Myo rest* ratio of hypoperfused myocardium at rest, *ACE* angiotensin-converting enzyme, *ARB* angiotensin receptor blocker, *CCB* calcium channel blocker, *Cr* creatinine, *CRP* C-reactive protein, *EDV* end diastolic volume, *eGFR* estimated glomerular filtration rate, *ESV* end systolic volume, *HbA1c* hemoglobin A1c, *HDL* high-density lipoprotein, *HRV* heart risk view, *LDL* low-density lipoprotein, *LVEF* left ventricular ejection fraction, *SPECT* single-photon emission computed tomography, *TG* triglyceride

### Follow-up MPS

**Fig. 1:**
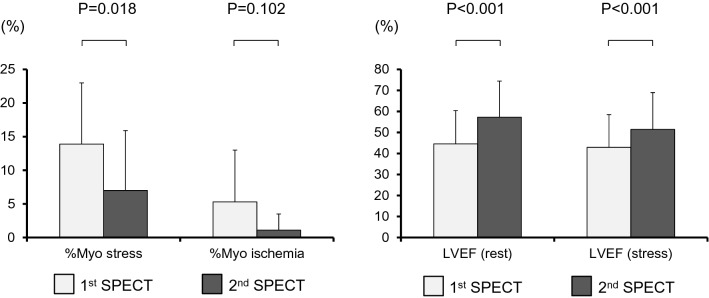
Changes in parameters among patients with ≥ 5% reduction of estimated risk of cardiac events. *%Myo ischemia* ratio of ischemic myocardium (from summed difference score), *%Myo stress* ratio of hypoperfused myocardium under stress, *LVEF* left ventricular ejection fraction, *SPECT* single photon emission computed tomography

**Fig. 2:**
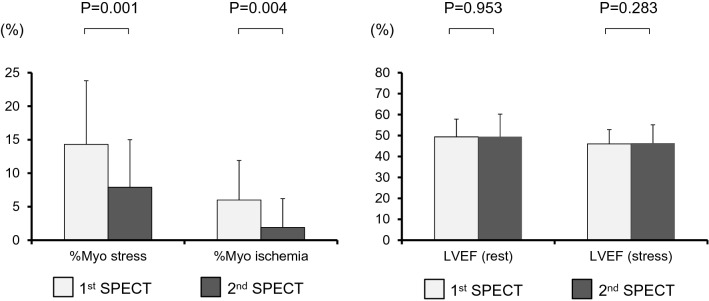
Changes in parameters among patients without ≥ 5% reduction of estimated risk of cardiac events. *%Myo ischemia* ratio of ischemic myocardium (from summed difference score), *%Myo stress* ratio of hypoperfused myocardium under stress, *LVEF* left ventricular ejection fraction, *SPECT* single photon emission computed tomography

Among 31 patients, HRV estimated ≥ 5% risk reduction in 14 of them. Table 2 shows the characteristics of patients with and without 5% estimated event risk reduction. Those with reduced event risk were older (*p* = 0.022), had a lower eGFR (*p* = 0.047) and were at higher event risk that at the first MPS assessment (*p* = 0.025). The %Myo stress, %Myo rest, and %Myo ischemia did not differ between the groups at the first MPS. The proportions of patients in both groups who were treated by revascularization between the first and second SPECT assessments were also identical. Table 3 shows a comparison between baseline and follow-up MPS data in patients with (A) and without (B) an estimated reduction in cardiac event risk (Figs. 1 and 2). The mean %Myo stress significantly decreased between baseline and follow-up (13.9 ± 9.1 vs. 7.0 ± 8.9, p = 0.018; 14.3 ± 9.5 vs. 7.9 ± 7.1, *p* = 0.001, respectively) in both groups. The LVEF at rest was significantly increased at the second, compared with the first study in patients with, but not in those without ≥ 5% risk reduction (57 ± 17 vs. 45 ± 16%, *p* < 0.001 and 50 ± 11 vs. 49 ± 9%, *p* = 0.953, respectively)**.**

**Table 2:** Comparison of patients with and without 5% reduction of estimated event risk

	Risk reduction ≥ 5% (*n* = 14)	Risk reduction < 5% (*n* = 17)	*p*
Age, years	79.6 ± 6.7	72.9 ± 8.4	0.022*
Male gender, *n* (%)	11 (79%)	14 (82%)	1.000
Cardiac risk factors, *n* (%)			
Hypertension	14 (100%)	13 (76%)	0.108
Diabetes	11 (79%)	17 (100%)	0.081
Dyslipidemia	10 (71%)	15 (88%)	0.370
Peripheral arterial disease	1 (7%)	1 (6%)	1.000
Current smoking	2 (14%)	4 (24%)	0.663
Family history	1 (7%)	0 (0%)	0.467
Blood findings			
CRP, mg/dL	0.3 ± 0.4 0.15 (0.04–0.3)^‡^	0.3 ± 0.6 0.1 (0.08–0.3)^‡^	0.874^†^
HbA1c, %	6.6 ± 0.9	6.9 ± 1.2	0.559*
Cr, mg/dL	1.1 ± 0.4	0.9 ± 0.2	0.043*
eGFR, mL/min/1.73 m^2^	48.9 ± 21.4 45 (39–62)^‡^	65.3 ± 19.4 64 (54–71)^‡^	0.047^†^
LDL cholesterol, mg/dL	100.6 ± 18.9	93.0 ± 23.8	0.351*
HDL cholesterol, mg/dL	49.1 ± 17.0 40 (37–59)^‡^	45.7 ± 13.0 42 (36–53)^‡^	0.691^†^
TG, mg/dL	117.0 ± 49.4 105 (86–152)^‡^	114.7 ± 58.5 109 (72–127)^‡^	0.648^†^
Medications, *n* (%)			
ACE inhibitors	3 (21%)	4 (24%)	1.000
ARB	6 (43%)	5 (29%)	0.707
CCB	7 (50%)	5 (29%)	0.457
Beta blockers	3 (21%)	7 (41%)	0.260
Nitrate	6 (43%)	5 (29%)	0.707
Aspirin	9 (64%)	12 (71%)	0.694
Clopidogrel	5 (36%)	6 (35%)	1.000
Statin	8 (57%)	13 (76%)	0.236
Insulin	2 (14%)	4 (24%)	0.657
CAG, *n* (%)	13 (93%)	17 (100%)	0.452
0 vessels	0 (0%)	1 (6%)	
1	4 (29%)	6 (35%)	
2	1 (7%)	5 (29%)	
3	8 (57%)	5 (29%)	
LMT lesion	2 (14%)	0 (0%)	0.179
1st SPECT			
%Myo stress	13.9 ± 9.1 18 (4–20)^‡^	14.3 ± 9.5 13 (9–19)^‡^	0.901^†^
> 5%, *n* (%)	10 (71%)	15 (88%)	0.370
> 10%, *n* (%)	9 (64%)	12 (71%)	1.000
%Myo rest	8.6 ± 9.4 5 (0.7–15)^‡^	8.3 ± 6.8 9 (3–12)^‡^	0.764^†^
> 5%, *n* (%)	7 (50%)	12 (71%)	0.288
> 10%, *n* (%)	5 (36%)	7 (41%)	1.000
%Myo ischemia	5.3 ± 7.7 0.7 (0–11)^‡^	6.0 ± 5.9 6 (0–12)^‡^	0.664^†^
> 5%, *n* (%)	5 (36%)	9 (53%)	0.473
> 10%, *n* (%)	4 (29%)	6 (35%)	1.000
LVEF, % (rest)	44.6 ± 15.9	49.4 ± 8.5	0.321*
EDV, mL (rest)	121.3 ± 61.7 112 (71–148)^‡^	113.4 ± 35.5 106 (86–144)^‡^	0.781^†^
ESV, mL (rest)	75.0 ± 59.0 57 (31–97)^‡^	59.6 ± 27.1 61 (39–76)^‡^	0.796^†^
LVEF, % (stress)	42.9 ± 16.0	46.0 ± 6.8	0.503*
EDV, mL (stress)	126.3 ± 55.0 118 (84–160)^‡^	111.8 ± 29.5 109 (93–135)^‡^	0.603^†^
ESV, mL (stress)	78.7 ± 53.4 62 (37–112)^‡^	61.4 ± 20.6 64 (45–79)^‡^	0.724^†^
2nd SPECT			
%Myo stress	7.0 ± 8.9 3 (0–10)^‡^	7.9 ± 7.1 6 (2–12)^‡^	0.467^†^
> 5%, *n* (%)	6 (43%)	9 (53%)	0.722
> 10%, *n* (%)	5 (36%)	7 (41%)	1.000
%Myo rest	6.0 ± 8.1 2 (0–10)^‡^	6.0 ± 5.2 4 (2–9)^‡^	0.492^†^
> 5%, *n* (%)	5 (36%)	8 (47%)	0.717
> 10%, *n* (%)	4 (29%)	4 (24%)	1.000
%Myo ischemia	1.1 ± 2.4 0 (0–3)^‡^	1.9 ± 4.3 0 (0–2)^‡^	0.877^†^
> 5%, *n* (%)	1 (7%)	3 (18%)	0.607
> 10%, *n* (%)	0 (0%)	1 (6%)	1.000
LVEF, % (rest)	57.3 ± 17.2	49.5 ± 10.8	0.133*
EDV, mL (rest)	105.7 ± 52.1 82 (69–139)^‡^	106.1 ± 32.4 113 (74–134)^‡^	0.606^†^
ESV, mL (rest)	51.9 ± 45.6 33 (21–67)^‡^	56.3 ± 25.9 61 (32–75)^‡^	0.258^†^
LVEF, % (stress)	51.4 ± 17.6	46.3 ± 8.8	0.335*
EDV, mL (stress)	110.7 ± 51.8 88 (78–137)^‡^	109.6 ± 31.2 118 (81–134)^‡^	0.592^†^
ESV, mL (stress)	59.9 ± 47.1 42 (29–82)^‡^	60.8 ± 24.8 63 (40–77)^‡^	0.427^†^
1st SPECT Risk estimated by HRV, %	20.8 ± 9.6 17 (14–26)^‡^	14.1 ± 4.2 13 (12–14)^‡^	0.025^†^
2nd SPECT Risk estimated by HRV, %	12.2 ± 9.1 8 (6–17)^‡^	13.0 ± 7.8 10 (9–13)^‡^	0.292^†^
Cardiac events, *n* (%)	0 (0%)	1 (6%)	1.000
Revascularization, *n* (%)	10 (71%)	12 (71%)	1.000

*%Myo ischemia* ratio of ischemic myocardium (from summed difference score), *%Myo stress* ratio of hypoperfused myocardium under stress, *%Myo rest* ratio of hypoperfused myocardium at rest, *ACE* angiotensin-converting enzyme, *ARB* angiotensin receptor blocker, *CAG* coronary angiography, *CCB* calcium channel blocker, *Cr* creatinine, *CRP* C-reactive protein, *EDV* end diastolic volume, *eGFR* estimated glomerular filtration rate, *ESV* end systolic volume, *HbA1c* hemoglobin A1c, *HDL* high-density lipoprotein, *HRV* heart risk view, *LDL* low-density lipoprotein, *LMT* left main trunk, *LVEF* left ventricular ejection fraction, *SPECT* single-photon emission computed tomography, *TG* triglyceride

*t Test

^†^*U* test

^‡^Median (25th–75th percentiles)

**Table 3:** Comparison of MPS data between baseline and follow-up in patients with (A) and without (B) reduced estimated risk of cardiac events

	1st SPECT	2nd SPECT	*p*
A: risk reduction ≥ 5%			
Risk estimated by HRV, %	20.8 ± 9.6 17 (14–26) ^‡^	12.2 ± 9.1 8 (6–17)^‡^	< 0.001^†^
%Myo stress	13.9 ± 9.1 18 (4–20) ^‡^	7.0 ± 8.9 3 (0–10)^‡^	0.018^†^
%Myo ischemia	5.3 ± 7.7 0.7 (0–11)^‡^	1.1 ± 2.4 0 (0–3)^‡^	0.102^†^
LVEF (rest), %	44.6 ± 15.9	57.3 ± 17.2	< 0.001*
LVEF (stress), %	42.9 ± 15.6	51.4 ± 17.6	< 0.001*
B: no risk reduction			
Risk estimated by HRV, %	14.1 ± 4.2 13 (12–14) ^‡^	13.0 ± 7.8 10 (9–13)^‡^	0.089^†^
%Myo stress	14.3 ± 9.5 13 (9–19) ^‡^	7.9 ± 7.1 6 (2–12)^‡^	0.001^†^
%Myo ischemia	6.0 ± 5.9 6 (0–12)^‡^	1.9 ± 4.3 0 (0–2)^‡^	0.004^†^
LVEF (rest), %	49.4 ± 8.5	49.5 ± 10.8	0.953*
LVEF (stress), %	46.0 ± 6.8	46.3 ± 8.8	0.283*

*%Myo ischemia* ratio of ischemic myocardium (from summed difference score), *%Myo stress* ratio of hypoperfused myocardium under stress, *HRV* heart risk view, *LVEF* left ventricular ejection fraction, *MPS* ^99m^Tc-tetrofosmin myocardial perfusion SPECT, *SPECT* single photon emission computed tomography

*t Test

^†^*U* test

^‡^Median (25th–75th percentiles)

### Left ventricular function

**Fig. 3:**
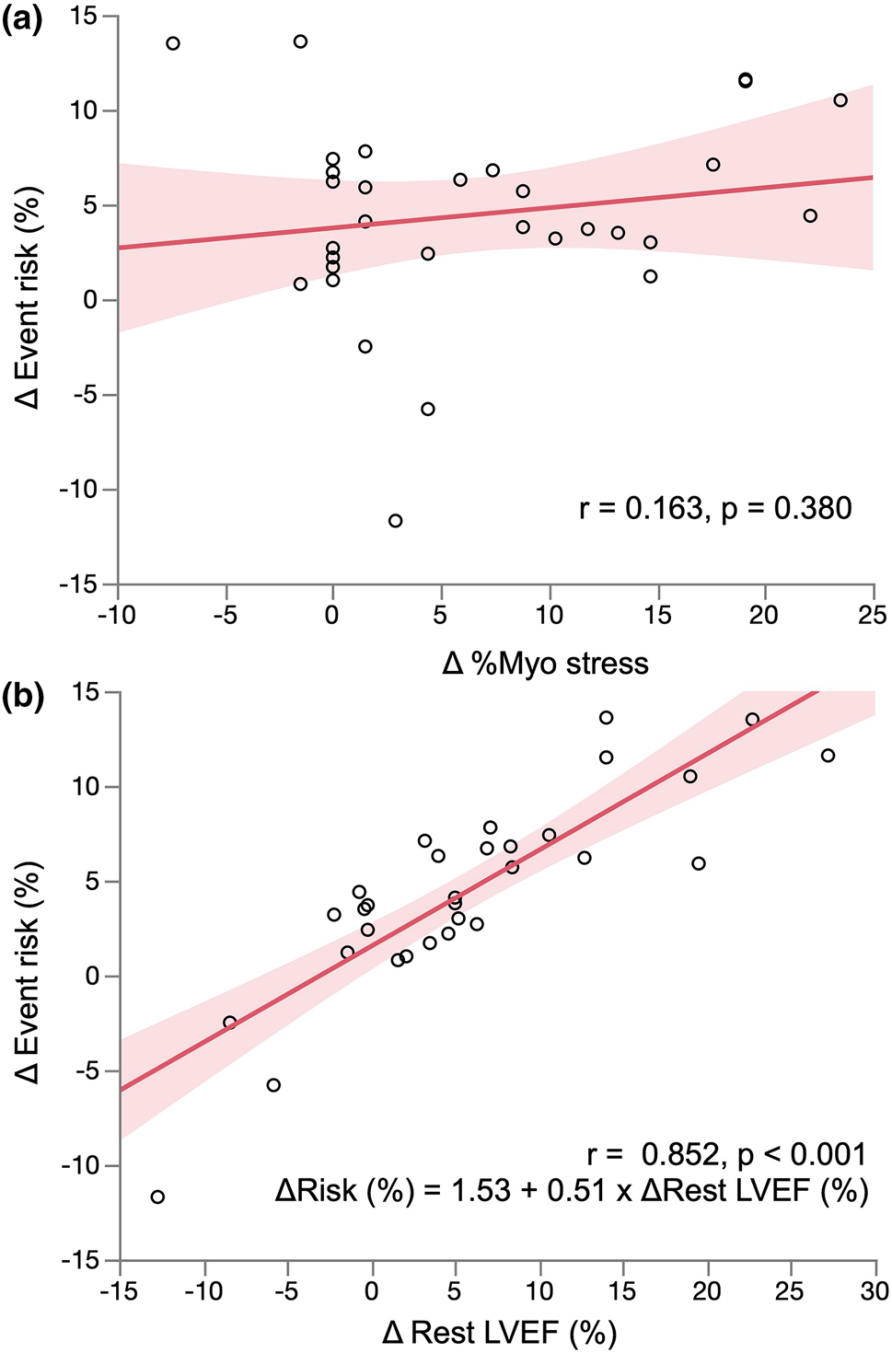
Correlations between ∆event risk estimated by HRV and ∆%Myo stress (**a**); ∆event risk and ∆rest LVEF (**b**). Shaded area, confidence level of fit. *%Myo stress* ratio of hypoperfused myocardium under stress, *HRV* Heart Risk View, *LVEF* left ventricular ejection fraction

**Fig. 4:**
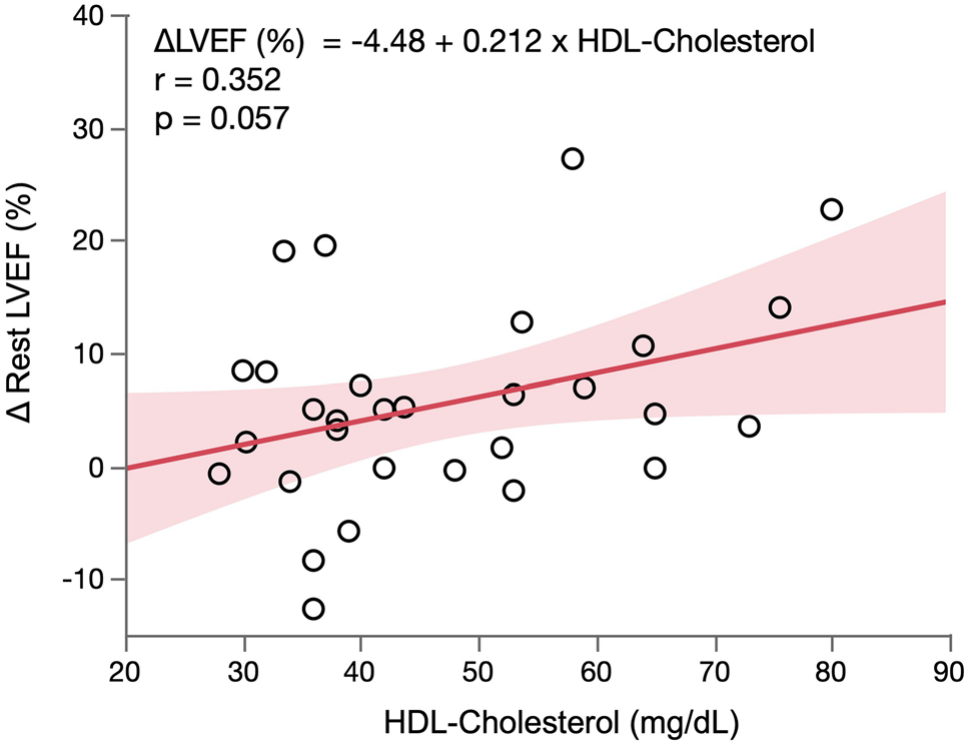
Correlation between ∆rest LVEF and HDL-cholesterol. Shaded area, confidence level of fit. *HDL* high-density lipoprotein, *LVEF* left ventricular ejection fraction

Since a decrease in estimated cardiac event risk depended on an increase in LVEF after PCI and/or OMT, the patients were separated according to whether or not they had a ≥ 10% increase in rest LVEF. Eight of 31 patients had ≥ 10% increase in rest LVEF. Table 4 compares the characteristics and MPS data between the patients with and without ≥ 10% increase in rest LVEF. Although the proportions of patients treated with statins did not significantly differ, HDL-cholesterol was significantly higher in patients with ≥ 10% improvement in rest LVEF (57 ± 16 vs. 44 ± 12 mg/dL, *p* = 0.032). The proportions of patients treated by PCI did not significantly differ between these groups. Figure 3 shows correlations between change in risk estimated by HRV and risk factors. The degree of improvement in event risk (∆event risk) did not correlate with the degree of improvement in %Myo stress (∆%Myo stress) (*r* = 0.163, *p* = 0.380) (Fig. 3a). However, ∆event risk significantly and positively correlated with ∆rest LVEF (*r* = 0.852, *p* < 0.001) (Fig. 3b). ∆Rest LVEF tended to positively correlate with HDL-cholesterol (*r* = 0.352, *p* = 0.057) (Fig. 4).

**Table 4:** Comparison of patients with and without 10% increase in LVEF

	LVEF ≥ 10% (*n* = 8)	LVEF < 10% (*n* = 23)	*p*
Age, years	78.5 ± 7.1	75.0 ± 8.3	0.309*
Male gender *n* (%)	5 (63%)	20 (87%)	0.160
Cardiac risk factors, *n* (%)			
Hypertension	8 (100%)	20 (87%)	0.549
Diabetes	6 (75%)	22 (96%)	0.155
Dyslipidemia	5 (63%)	20 (87%)	0.160
Peripheral arterial disease	0 (0%)	2 (9%)	1.000
Current smoker	1 (13%)	5 (22%)	1.000
Family history (cardiac diseases)	1 (13%)	0 (0%)	0.267
Blood examination			
CRP, mg/dL	0.2 ± 0.2 0.08 (0.04–0.2)^‡^	0.4 ± 0.5 0.19 (0.07–0.3)^‡^	0.309^†^
HbA1c, %	6.6 ± 0.5	6.8 ± 1.1	0.624*
Cr, mg/dL	1.0 ± 0.4	1.0 ± 0.3	0.810*
eGFR, mL/min/1.73 m^2^	56.7 ± 23.8	59.9 ± 19.6	0.716*
LDL cholesterol, mg/dL	97.3 ± 16.6	96.0 ± 22.7	0.894*
HDL cholesterol, mg/dL	57.4 ± 16.4	44.0 ± 12.1	0.032*
TG, mg/dL	124.0 ± 42.7 105 (96–136)^‡^	112.9 ± 55.6 109 (71–138)^‡^	0.429^†^
Medications, *n* (%)			
ACE inhibitors	2 (25%)	5 (22%)	1.000
ARB	4 (50%)	7 (30%)	0.412
CCB	6 (75%)	6 (26%)	0.034
Beta blockers	2 (25%)	8 (35%)	0.682
Nitrate	3 (38%)	8 (35%)	1.000
Aspirin	6 (75%)	15 (65%)	1.000
Clopidogrel	2 (25%)	9 (39%)	0.672
Statin	3 (38%)	18 (78%)	0.074
Insulin	1 (13%)	5 (22%)	1.000
CAG			
Vessel, *n*	2.0 ± 0.9	2.0 ± 1.0	0.897
Multivessel, *n* (%)	3 (38%)	8 (35%)	1.000
Left main, *n* (%)	0 (0%)	2 (9%)	1.000
1st SPECT			
%Myo stress	13.9 ± 9.9	14.2 ± 8.7	0.916*
%Myo rest	9.6 ± 10.4 5.2 (0–18)^‡^	8.1 ± 6.7 7.4 (3.0–12)^‡^	0.945^†^
%Myo ischemia	4.2 ± 7.9 0 (0–5)^‡^	6.1 ± 5.9 5.9 (0–12)^‡^	0.339^†^
LVEF, % (rest)	43.2 ± 11.3	48.6 ± 12.2	0.297*
EDV, mL (rest)	120.3 ± 38.1 130 (79–152)^‡^	115.8 ± 50.3 105 (79–145)^‡^	0.619^†^
ESV, mL (rest)	71.8 ± 33.9 74 (39–96)^‡^	65.9 ± 47.0 58 (32–79)^‡^	0.372^†^
LVEF, % (stress)	42.5 ± 11.6	45.3 ± 11.0	0.565*
EDV, mL (stress)	127.8 ± 36.6 140 (89–162)^‡^	115.2 ± 43.9 109 (86–140)^‡^	0.222^†^
ESV, mL (stress)	77.1 ± 34.4 82 (40–107)^‡^	66.7 ± 40.2 62 (39–82)^‡^	0.324^†^
2nd SPECT			
%Myo stress	7.0 ± 10.0	7.7 ± 7.2	0.833*
%Myo rest	5.3 ± 8.9 0.7 (0–6)^‡^	6.2 ± 5.9 4 (0.7–10)^‡^	0.752^†^
%Myo ischemia	1.7 ± 2.7 0 (0–3)^‡^	1.5 ± 3.9 0 (0–2)^‡^	0.909^†^
LVEF, % (rest)	60.7 ± 14.5	50.3 ± 13.6	0.077*
EDV, mL (rest)	104.3 ± 40.4 97 (74–144)^‡^	106.5 ± 43.0 110 (71–133)^‡^	0.883†
ESV, mL (rest)	44.9 ± 27.3 46 (27–63)^‡^	57.6 ± 38.0 55 (30–77)^‡^	0.394^†^
LVEF, % (stress)	54.0 ± 15.1	46.8 ± 12.7	0.200*
EDV, mL (stress)	109.5 ± 42.0 103 (77–146)^‡^	110.4 ± 41.6 110 (80–133)^‡^	0.961^†^
ESV, mL (stress)	54.6 ± 31.9 55 (35–73)^‡^	62.4 ± 39.6 61 (36–78)^‡^	0.604^†^
Risk estimated by HRV, %	19.6 ± 7.9 17 (14–23)^‡^	16.3 ± 7.4 13 (12–16)^‡^	0.161^†^
Change in estimated risk by HRV ≥ 5%, *n* (%)	8 (100%)	6 (26%)	< 0.001
Revascularization, *n* (%)	5 (63%)	17 (74%)	0.659

*%Myo ischemia* ratio of ischemic myocardium (from summed difference score), *%Myo stress* ratio of hypoperfused myocardium under stress, *%Myo rest* ratio of hypoperfused myocardium at rest, *ACE* angiotensin-converting enzyme, *ARB* angiotensin receptor blocker, *CAG* coronary angiography, *CCB* calcium channel blocker, *Cr* creatinine, *CRP* C-reactive protein, *EDV* end diastolic volume, *eGFR* estimated glomerular filtration rate, *ESV* end systolic volume, *HbA1c* hemoglobin A1c, *HDL* high-density lipoprotein, *HRV* heart risk view, *LDL* low-density lipoprotein, *LVEF* left ventricular ejection fraction, *SPECT* single-photon emission computed tomography, *TG* triglyceride

*t Test

^†^*U* test

^‡^Median (25th–75th percentiles)

### Cardiac events during follow-up

One patient who did not have a ≥ 5% estimated risk reduction and who was not treated by PCI was hospitalized due to heart failure within a follow-up of 22.1 ± 6.7 months.

## Discussion

We prospectively followed-up patients who were estimated by HRV as being at moderate-to-high risk of cardiac events. Reducing estimated risk mostly depended on decreasing %Myo stress and increasing LVEF; that is, a subsequent reduction in cardiac ischemia and increase in LVEF brought about by revascularization and/or OMT.

Patients at low risk such as those with stable CAD derived no benefit of PCI over OMT [4], whereas high-risk patients with moderate-to-severe ischemia had a poor prognosis when revascularization was not added to OMT [8–10]. The ISCHEMIA trial revealed that coronary revascularization in patients with stable CAD and moderate or severe ischemia does not reduce the likelihood of ischemic cardiovascular events compared with OMT [17]. However, in the late follow-up phase of that study, cardiac events occurred more frequently in patients treated by conservative medical therapy than by invasive therapy. The results indicated that ischemic burden alone at a single time point was insufficient to determine treatment plans for patients at moderate-to-high risk. In addition to the initial choice of treatment strategy, re-assessment of the clinical course during the follow-up period was considered equally important. Therefore, we quantitatively evaluated cardiac risk using HRV software and determined whether treatment reduced moderate-to-high cardiac risk.

Normal stress MPS findings in the Japanese population are associated with low cardiac event risk [18]. However, cardiac events develop in 3% of patients with CAD at higher estimated cardiac risk even when stress MPS findings are normal [19]. Although several studies have concluded that %Myo stress is a clinical indicator [10, 11], risk estimation by HRV, which integrates several risk factors, could be more useful than %Myo stress for predicting cardiac events [14, 15], especially in patients with moderate-to-high risk. In our study, the risk estimated by HRV in the first SPECT was significantly higher in patients with 5% reduction in estimated event risk, although %Myo stress, LVEF, and the proportions of patients with diabetes did not differ between two groups at the first MPS (Table 2). This is not surprising because risk estimation by HRV depended on composite effects of multiple risk factors. In other words, even though each parameter did not have a significant difference, the risk estimated by HRV might have significant differences between two groups. We recommend a two-step approach for such patients as follows. The ratio (%) of the ischemic burden should be assessed if patients have high %Myo stress. Cardiac event rates were significantly lower among patients who achieved ≥ 5% reduction in ischemia after revascularization and/or OMT compared with those who did not [5–7]. Accordingly, if patients had ≥ 5% myocardial ischemia at the initial assessment, revascularization should be indicated. Second, if patients had low %Myo stress with moderate-to-high risk estimated by HRV, cardiac ischemia should be assessed using other modalities because MPS results can be false-negative, for example, in patients with left main CAD [20]. In our study, ∆event risk and ∆%Myo stress did not correlate (Fig. 3a). The %Myo rest was relatively high in the present study (8.4 ± 7.9%), even though we excluded patients with a prior diagnosis of myocardial infarction. This might be based on the characteristics of the patients, because we included several with relatively severe ischemia. Even if the patients had high %Myo stress, revascularization did not always reduce %Myo stress. The severity of cardiac ischemia in these patients could have been underestimated if judged only by ischemic burden. Thus, integrated risk estimation using HRV, in addition to %Myo ischemia, was useful to decide treatment policies. Therapy guided by estimated risk facilitated assessments of cardiac event probability and even treatment effects. When cardiac event risk estimated by HRV does not decrease sufficiently even after intensive therapies, interventions other than coronary revascularization and OMT, such as lifestyle changes and nutrition counselling, should be applied.

Only one patient experienced a cardiac event during follow-up in the present study. One reason for the low cardiac event rate was due to relatively small sample size (*n* = 31) and < 3 years (22.1 ± 6.7 months) of follow-up. A larger sample and a longer follow-up might have resulted estimations of cardiac event risk that were comparable to real cardiac events. However, a previous evaluation of cardiac risk using the predictive value of the same J-ACCESS risk model used herein, confirmed essentially the same characteristics in 283 patients [15]. We concluded that a reduction in cardiac ischemia and an increase in LVEF by revascularization and/or OMT were both needed to reduce the likelihood of cardiac events. This was anticipated, because risk was estimated by the J-ACCESS model using several risk factors including LVEF and %Myo stress. Thus, our conclusion might be reasonable irrespective of sample size. The LVEF did not improve in several patients, although %Myo stress decreased. As described above, ∆event risk did not correlate with ∆%Myo stress, but ∆event risk significantly correlated with ∆rest LVEF. Our findings concurred with the previous reports [14, 15, 17].

We found an association between improved LVEF and high serum HDL-cholesterol values. A reduction in estimated risk mostly depended on a decrease in %Myo stress and an increase in LVEF. High HDL-cholesterol contributed to the improvement in cardiac function after therapy, and a subsequent reduction in cardiac event risk determined by HRV. Estimated risk did not significantly change after treatment in half of our patients who achieved a reduction in %Myo stress, because LVEF did not improve after therapy. Low serum HDL-cholesterol values comprise an independent predictor of cardiac events in patients with CAD [21–23]. High serum total cholesterol values are also associated with worse outcomes in patients with congestive heart failure due to CAD [24]. Statin therapy caused a regression in coronary atherosclerosis when LDL-cholesterol was reduced, and HDL-cholesterol was increased by > 7.5% [25]. Levels of HDL-cholesterol inversely correlate with atherosclerotic progression [26]. Myocardial damage induced by PCI is more prevalent in patients with lower HDL-cholesterol levels, and HDL-cholesterol is an important cardio-protective factor in patients undergoing coronary revascularization [27]. An experimental model has shown that HDL-cholesterol reduces plaque lipid content and increases proportions of collagen and smooth muscle cells [28]. These effects could reduce the likelihood of coronary arterial embolization during PCI. We found that ∆rest LVEF weakly and positively correlated with HDL-cholesterol levels. One reason might be that PCI was applied to only 64% of the patients with > 10% increase in LVEF (Table 4). Thus, high HDL-cholesterol levels contributed to the improved cardiac function, especially after PCI and led to reduced cardiac event risk estimated by HRV.

### Limitations

Only one patient experienced a cardiac event in this small cohort. The mean follow-up period was 22.1 ± 6.7 months, but the HRV calculated cardiac event probability for three years. In 17 patients without ≥ 5% reduction in estimated cardiac risk, the mean estimated risk at 3 years after treatment was 13.0% ± 7.8%, and the patient with the cardiac event was in this group (event rate 5.8%). However, the HRV estimation of future cardiac events was essentially within a suitable range.

## Conclusions

A reduction in cardiac ischemia and an increase in LVEF by revascularization and/or OMT were needed to reduce the likelihood of cardiac events in patients who were estimated by HRV as being at moderate-to-high risk. Low serum HDL-cholesterol was associated with less improvement of LV function, which resulted in insufficient improvement of risk estimated by HRV. Patients who have moderate-to-high cardiac event risk estimated by HRV even after treatment should be more carefully managed, especially when cardiac dysfunction persists.
